# Advanced Strategies of Drug Delivery via Oral, Topical, and Parenteral Administration Routes: Where Do Equine Medications Stand?

**DOI:** 10.3390/pharmaceutics15010186

**Published:** 2023-01-04

**Authors:** Yunmei Song, Candace M. Day, Franklin Afinjuomo, Jin-Quan E. Tan, Stephen W. Page, Sanjay Garg

**Affiliations:** 1 Centre for Pharmaceutical Innovation (CPI), Clinical and Health Sciences, University of South Australia, Adelaide, SA 5000, Australia; 2 SA Pharmacy, Flinders Medical Centre, Southern Adelaide Local Health Network, Adelaide, SA 5042, Australia; 3 Advanced Veterinary Therapeutics, Newtown, NSW 2042, Australia

**Keywords:** equine, oral, topical, injection, NDDSs

## Abstract

While the global market for veterinary products has been expanding rapidly, there is still a lack of specialist knowledge of equine pharmaceutics. In many cases, the basic structure of the gastrointestinal tract (GIT) and integumentary system of the horse shares similarities with those of humans. Generally, the dosage form developed for humans can be repurposed to deliver equine medications; however, due to physiological variation, the therapeutic outcomes can be unpredictable. This is an area that requires more research, as there is a clear deficiency in literature precedence on drug delivery specifically for horses. Through a careful evaluation of equine anatomy and physiology, novel drug delivery systems (NDDSs) can be developed to adequately address many of the medical ailments of the horse. In addition to this, there are key considerations when delivering oral, topical, and parenteral drugs to horses, deriving from age and species variation. More importantly, NDDSs can enhance the duration of action of active drugs in animals, significantly improving owner compliance; and ultimately, enhancing the convenience of product administration. To address the knowledge gap in equine pharmaceutical formulations, this paper begins with a summary of the anatomy and physiology of the equine gastrointestinal, integumentary, and circulatory systems. A detailed discussion of potential dosage-form related issues affecting horses, and how they can be overcome by employing NDDSs is presented.

## 1. Introduction

Horses are companion animals with temperamental and sensitive characteristics that play an important role in the lives of many individuals. Therefore, the global demand and market for equine healthcare products are rapidly increasing. However, comprehensive, and intensive information on drug development relating to equine health is still very much in its infancy. Delivering drugs to horses orally is a major challenge, as the physiology and anatomy of the equine gastro-intestinal tract (GIT) can result in poor bioavailability of some oral drugs. Additionally, due to their size, the dosages required are much larger and higher cost compared to those of humans; with potential of adverse effects, especially for antibiotics, following the administration of some drugs [1]. There are also differences between various breeds, and ages (from foals to mature adults), resulting in more diverse clinical requirements [1]. Unfortunately, there is no generalized treatment that is suitable for all horses, as they may respond differently to the same treatment [2]. Therefore, a thorough examination of each horse is required with/without an adjustment of the therapy regimen [2]. These challenges further limit the selection of drugs for equine treatment. Moreover, due to the lack of literature precedence, together with the limited understanding and research efforts in medical technology specifically for equines, the diagnosis of their diseases can be slow, with low treatment success rates and extended recovery times. For example, Mitchell et al. reported that insufficient knowledge and understanding of the pathophysiology of laminitis, inadequate efforts for prevention and therapy, and the absence of specific treatments for laminitis resulted in significant financial losses and emotional tension for owners [2]. Therefore, timely diagnosis and better treatment options are essential to ensure a higher likelihood of therapeutic success [2].

There is recent increased interest in novel pharmaceutical technologies applicable to equine medications. These technologies will support the improvement of equine health and well-being, through better treatment approaches. Apart from effective medications, owners need to be educated to understand the benefits of the latest medical technology, and they must be willing to accept the higher cost of new treatments. Veterinarians and owners play critical roles in supporting the best treatments for equine health and welfare. To the best of our knowledge, the key parameters concerning the development of NDDs intended specifically for equines have not been previously reviewed.

To address the knowledge gap and deficiency in literature precedence, as well as to expand the knowledge pool in equine health, this paper aims to provide thorough and detailed insights into the anatomy and physiology of the equine gastrointestinal system, skin structure, and injection sites. Most importantly, a detailed discussion of recent advancements in NDDSs and key considerations to be considered in new drug developed specifically for horses will follow. Finally, we will provide our insights into the challenges and future trends in equine product development.

## 2. Equine Pharmacology

The use of therapeutic molecules is particularly challenging in the horse. Unlike small animals such as dogs which often act as prototypical species for basic pharmacological research before an intended use in humans; equines are not commonly incorporated in pre-clinical testing during drug-development, leading to a lack of data regarding therapeutic molecules for the treatment of different diseases in equines [3,4]. Additionally, the limited understanding of the impact of equine anatomy and physiology on new drugs directly contributes to the uncertainty and unpredictability of potentially therapeutically active molecules [5].

### 2.1. Preferred Route of Administration

Generally speaking, oral administration of medications is the most common and preferred route [6,7] due to their convenience and they should be prioritized if therapeutically appropriate. In addition to this, the GIT has the a natural immunological and physical defense mechanism to cope with foreign materials [5,6]. Moreover, the cumulative cost of formulating an oral drug is much less and more convenient, compared to the preparation of a sterile parenteral product [5,8].

One of the most important factors to be considered when formulating an oral administration is the bioavailability of therapeutic molecules [8]. There is a large body of evidence demonstrating that for many drug substances oral bioavailability is relatively acceptable in mono-gastric species but much less so in the horse. For instance, several drugs including tramadol, furosemide, and acyclovir exhibit poor bioavailability as low as 10% in equines, as a result of poor absorption, despite being adequately absorbed in other species [5]. The oral bioavailability of medications can also be influenced by their feeding times. This is demonstrated through a study in which the poor oral bioavailability of ampicillin in horses was attributed to the extensive binding of ampicillin to hay, thereby, subsequently causing an increased residence time in the gastric acidity of horses during feeding, consequently reducing the drug stability and bioavailability [9]. While strategic fasting can be employed to avoid the deleterious effects of feeding on oral bioavailability, this can be very complicated in the cases of medications that are required to be dosed more than once daily [5].

### 2.2. Metabolism and Prodrugs in Horses

In addition to oral bioavailability, it is also important to consider the differences in equine metabolism and its implications on the administered medications. These differences might hinder the application and extrapolation of human medications and other species to horses [10]. An example is prednisone, a pro-drug that is bio-activated to prednisolone by hepatic enzyme 11-β-hydroxy-dehydrogenase [11]. Unlike dogs and humans, horses do not readily convert prednisone to its active form, as prednisone is poorly absorbed, and it is inactive in its natural form when administrated to horses [5].

### 2.3. Parenteral Route of Administration

Parenteral routes, including intravenous (IV), subcutaneous (SC), and intramuscular (IM) injections, are used when the horse is unable to absorb the orally administered drug adequately, due to reduced perfusion, shock, or suffering from colic [12]. It also provides a means to overcome the first-pass metabolism, providing rapid onset of action if administered intravenously [12]. However, with IV administration, without proper training in injection technique, there is a risk of accidental injection into the carotid artery rather than the jugular vein, resulting in severe Central Nervous System (CNS) effects such as seizures [5,13].

Intramuscular (IM) injections can be performed to achieve a prolonged duration of action, using a depot formulation [14,15]. Depot formulations are appropriate for medications such as prednisolone a corticosteroid, which are also used in joints for their anti-inflammatory effect [16]. Common sites for IM injections include the neck, rump, and pectoral muscles; ideally, the site of injection should be rotated if frequent administration is required, noting that the pharmacokinetic profiles of IM drugs may vary according to the site of administration [5,17,18]. However, IM administration of several drugs has been linked to clostridial myositis, including with the Nonsteroidal anti-inflammatory drug (NSAID) flunixin meglumine [19]. Thus, special precautions and care are recommended when administering drugs intramuscularly; and the IM administration of several medications including NSAIDs should be avoided when possible [20].

According to Equine Formulary, there are 18 actives that can be given SC. However, compared to IV and IM sites, SC route is less often employed as a site of injection, and compounds which cause irritations should not be given through SC route to prevent tissue damage [5,6].

In addition to the primary parenteral routes of administration such as IV, IM and SC, there are other application-specific subtypes of parenteral administration such as intra-articular, intracardiac, intra-arterial, intrathecal or peridural. However, with the lack of in-depth literature precedence, the application of these routes in drug delivery is still very much in its infancy. Besides these routes, regional limb approaches for antibiotic or analgesia are a growing interest, though no products are specifically registered for this route. Where a peripheral vessel is accessible, an effective tourniquet can be applied to isolate the infected region. In this case, Intraosseous (IORLP) or intraarticular (IARLP) perfusion will also provide a high concentration of antibiotic at the regional site of infection [21].

### 2.4. Pharmacokinetic and Pharmacodynamic Drug Interactions

The nature of drug interactions can be classified as pharmacokinetic or pharmacodynamic. Pharmaco-dynamic interactions can either be beneficial or detrimental. Therefore, it is important to cautiously administer a combination of medications, especially if there is a lack of clinical evidence of safety and efficacy. One of the examples of synergistic combinational antibiotics is the co-administration of gentamicin and penicillin, where penicillin can facilitate the access of gentamicin into the bacterial cells which subsequently results in enhanced bactericidal activity [22]. In contrast, when some cephalosporins such as cefotaxime and ceftriaxone are co-administrated with chloramphenicol, detrimental outcomes can result. For instance, it was reported in a study that co-administrating chloramphenicol at 8 µg/mL with cephalosporin resulted in a significant increase in the minimum bactericidal concentration (MBC) of Cephalosporin (0.28–21.5 µg/mL) [23]. Therefore, careful consideration of combination treatment is warranted.

The majority of drug interactions are related to metabolism by the cytochrome (CYP) P450 enzyme superfamily [24]. These interactions between drugs are triggered through either the induction or inhibition of enzymes involved in metabolism. Induction is usually caused by the upregulation of enzyme expression and synthesis of new enzymes [25]. Thus, the induction effect is usually time-consuming and delayed. In contrast, the enzymatic inhibition effect induces the clearance of a substrate metabolized by a specific enzyme, and this inhibiting effect often occurs immediately [26,27]. In most cases, these pharmacokinetic interactions can be overcome by altering the medication doses, ensuring that their required concentrations remain at an effective and safe range in the body [28].

Furthermore, the CYP P450 superfamily consists of various isoforms, and it is further divided into subfamilies based on their genotypic homology, including CYP1A, CYP3A, CYP2D, and CYP3C [29]. The CYP3A4 isoform is responsible for the metabolism of most therapeutic molecules in humans [30]. Unfortunately, the metabolism of therapeutics by CYP families is highly specific for each substrate and species; therefore, the metabolism data of xenobiotics metabolized by the CYP enzymes obtained in humans might not simply be extrapolated to other mammals, including horses [31,32,33,34]. Even though some equine P450 enzyme isoforms have been sequenced and expressed in recombinant systems [31,32,33,34], they are not widely available for routine testing. Consequently, there is a lack of data regarding pharmacokinetic drug interactions in equines, specifical data from the CYP enzymes [5,33].

### 2.5. Differences between Foals and Adult Horses

Like humans and many other animal species, as foals are actively growing, they are physiologically different from mature horses and are referred to as “moving” drug targets. As a result, evidence of drug use in mature adult horses should not be directly applied and extrapolated to foals. The key differences in adult horses and foals are therefore should be taken in account in formulating equine medications. The important factors to consider when treating foals are summarized in Table 1 below.

## 3. Oral Administration in Horse

### 3.1. Anatomy and Physiology of the Gastrointestinal Tract (GIT) of the Horse

For a neonatal horse, the weight of their GIT and liver is around 35 g/kg of total body weight [43]. The main function of the liver at this stage is the storage of nutrients. As the horse reaches six months of age, the weight of the GIT has increased to around 60 g/kg of total body weight [43]. The weight of the GIT eventually stabilizes at 45–60 g/kg when the horse reaches one year of age [43]. Interestingly, the size of the liver continues to decrease by approximately12–14 g/kg at six months and stabilizes at 10 g/kg at one year old [43].

Other factors can influence the weight of the GIT and the liver; however, the mechanisms for the changes in liver parameters are not fully understood. For example, the ingestion of food can readily change the size of the liver, as its size increases quickly after a meal, owing to increased glycogen production stored in the liver. This process requires more blood to flow through the liver, therefore, leading to an increase in the perfusion of the liver [43], resulting in an increase in postprandial liver size. The second factor contributing to the anatomical change of a horse’s anatomy is physical activity. When a human exercises, the perfusion to the GIT is reduced, and the blood flow is diverted to improve oxygen delivery to the muscles [44]. Therefore, the activity of GIT consequently is stabilized [45]. As an extension of the human experience, this phenomenon can also occur when a horse is trained/exercised [46].

**Figure 1:**
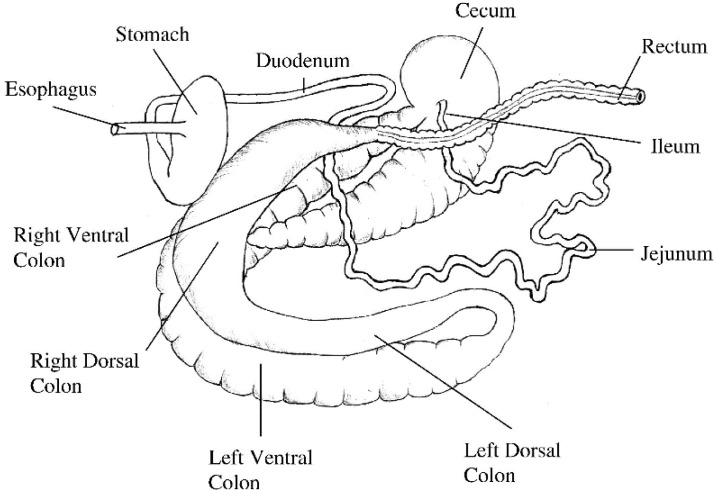
Isolated gastrointestinal tract of the adult horse. Image reprinted with permission from Van Weyenberg and Janssens [48]. 2022, Elsevier.

The length of the small intestine does not change significantly after four weeks of age; while the large intestine continues to grow until the horse reaches 20 years of age [43]. For a 500 kg adult horse, the length of the small intestine is approximately 16 m, the caecum is about 0.8 m, the ascending colon is 3 m and the descending colon is 2.8 m [43], and the relative capacity is approximately 30.2%, 15.9%, 38.4% and 7.0%, respectively (Figure 1) [47].

#### 3.1.1. Anatomy and Physiology of the Horse’s Stomach

For an adult horse, the stomach is relatively small compared to the GIT, only comprising approximately 8% of the GIT [49]. Most digesta are held in the stomach for 2–6 h only, however, the stomach is rarely empty. Liquid contents halve in volume after 30 min, and solid contents halve in volume after around 1.5 h [50]. At the same time, some digesta can pass to the duodenum when the ingesta reaches the stomach. This process stops when the horse stops eating and there is no more ingesta entering the stomach [50]. When the horse drinks water at the same time, the curvature of the stomach wall will induce the mixing of water with the digesta, assisting the dilution of digested components within the stomach [50,51,52,53,54].

#### 3.1.2. pH Value of the Horse Stomach

**Figure 2:**
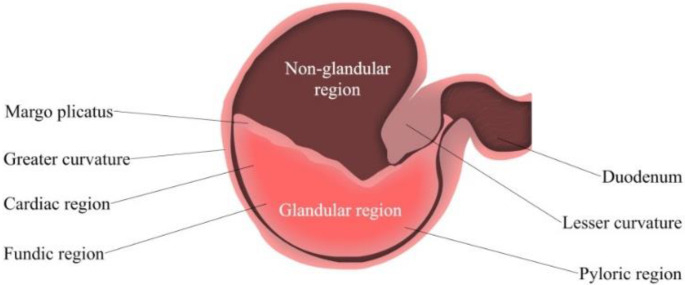
Simplified Isolated Stomach of the adult horse. From left to right: Fundic (upper region) and pyloric (lower region).

Gastric pH is determined by the gastric acid secretion and gastric contents [55]. The ventral portion of the equine stomach is lined by glandular mucosa; and the entire mucosa is separated into two parts, fundic and pyloric regions [56] (Figure 2). The fundic mucosa contains parietal cells and zymogen cells. Parietal cells can produce hydrochloric acid (HCl), while zymogen cells secrete pepsin. The pyloric region can produce the polypeptide hormone gastrin, and release it into the blood plasma [43]. This hormone secretion is typically triggered by a meal, simultaneously with the production of gastric acid and gastric juice for digestion. The pyloric region is the most acidic region of the stomach, followed by the glandular fundus and the squamous mucosa [50]. HCl is constantly secreted despite an empty stomach; this process is known as basal secretion. As reported by Merritt and Jullian, horses secrete approximately ~200 µeq/kg/h of HCl mixed with pancreatic and duodenal fluids [49].

The stomach pH value can be determined by various methods, such as gastric fluid aspiration, gastric cannulation, or by using a pH electrode [56]. In a previous study conducted by Murray and Grodinsky [57], the average pH of luminal contents was revealed to be 1.8 in the stomach, and 3.5 in the antrum [57]. However, other studies have shown that pH values can change and fluctuate in response to food. For example, Murray and Schusser demonstrated that the basal pH of a horse is highly acidic, and the acidity decreases after the horses were fed, causing fluctuations in stomach pH after a few hours, whereas in the case of sham feeding, the stomach pH increases. Conclusively, feeding, and sham feeding increase gastric acid secretions; while food consumption can provide a temporary buffer effect [56].

#### 3.1.3. Motility of Gastrointestinal Tract

Most of the digesta are held in the stomach for around 2 to 6 h [58]. When fresh ingesta enter the stomach, peristaltic contractions are initiated, and the existing digesta will move into the duodenum. However, when the ingestion stops, the contractions will decrease, hence, the movement of digesta into the duodenum will subsequently stop. Once they reach the duodenum, transit time is rapid in the small intestine with a rate of approximately 30 cm/min, and this rate is largely affected by the distance to the pylorus [3]. The further digesta is from the pylorus, the lesser the frequency of the contractions, hence, a longer transit time [48]. The digesta moves from the ileum to the cecum through the ileocecal valve which opens when the pressure in the ileum increases. This movement is initiated by the distention of the stomach and the increased motility of the ileum. Contents are then directed into the ceco-colic junction before arriving at the ventral colon, which is driven by the strong peristaltic contraction in the cecum. In most cases, the majority of the digest will reach the cecum and colon within 3 h of food consumption [48].

In the colon, there are 3 different types of contractions affecting the motility of the large intestine that are rhythmic contractions, that move in an aboral direction; another rhythmic contraction that propagates orally; and a third less intense isolated contraction that does not propagate in any direction but is responsible for 90–95% of the motility from the large intestine [48].

#### 3.1.4. Gastric Emptying Time

The gastric emptying times for materials of solid, liquid, and oil nature occur at different rates. Generally, liquids tend to exit the stomach much faster compared to other materials [59,60]. Several methods can be used to access the gastric emptying time, such as gastric radioscintigraphy, gastric ultrasonography, stable isotope breath test, Acetaminophen absorption test, and marker dilution techniques [47].

Gastric radioscintigraphy involves the ingestion of a test meal labeled with a known radionuclide of specific energy [61]. Once ingested, its movement through the intestinal tract is then monitored using a gamma camera. Despite being considered an advanced technique for assessing GIT motility, there are very few studies that employed gastric radioscintigraphy [47]. For example, through the use of gastric radioscintigraphy in the horse Bahr et al. [62] reported that the gastric emptying half time is 1.50 ± 0.17 h. This result is in line with the finding of Levy and Sojka [63], reporting a gastric half-emptying time of 1.50 ± 0.18 h.

The stable isotope breath test is another technique used to determine gastric emptying time [64]. It involves the measurement of the rate of increments in the expiration of ^13^C:^12^C ratio, following the ingestion of a meal labeled with a stable non-radioactive isotope, ^13^C [65]. The ^13^C:^12^C ratio can then be used as an indirect measure of the gastric emptying rate for that particular meal. Sutton conducted several breath tests in the horse to assess the gastric emptying of various solid and liquid materials [47]. In this study, bicarbonate and acetate breath tests were performed to assess the gastric emptying and gastric half emptying time of liquid materials, in which 1.13 ± 0.35 h and 1.72 ± 0.21 h were reported, respectively. However, it was also important to notice the difference in gastric half emptying time between the bicarbonate and acetate test. The reason for this is that the ^13^C-acetate requires time to be metabolized, hence, resulting in a slightly longer gastric emptying time. ^13^C-octanoic acid breath test was also conducted to assess the solid material gastric emptying and a gastric half emptying time, with a value of 3.79 ± 1.53 h observed [47].

Other factors that can influence the gastric emptying rate include the size of the meal and the nutritional content. In a study conducted by Métayer et al., it was reported that consuming a meal with low starch content increases gastric emptying time significantly [66]. Additionally, from this experiment, it was demonstrated that a calorie deficit and low-starch meal with high insoluble fiber content emptied faster, compared to a high-starch meal [66]. However, differences in caloric content did not have a significant influence on the gastric emptying rate. In addition to this, it was also revealed that larger meals have longer gastric emptying times, compared to smaller meals [66].

#### 3.1.5. Transit Time of GIT

Adequate control of digesta within the GIT is essential for proper digestion; otherwise, gastrointestinal disorders can occur. There are many processes needed for complete digestion following ingestion. For example, the GIT will mix the ingesta with the GI secretion; the ingesta is then hydrolyzed by digestive enzymes; non-digestible materials are then fermented by commensal bacteria within the GIT, producing by-products that can be absorbed [43]. Feed type is also another major factor that can influence GIT transit time [67,68], especially the rate at which the digesta passes through the small intestine. It is found that high-fiber diets have a much shorter transit time compared to low-fiber diets of the same size [43].

##### Whole Gut Transit Time

Whole gut transit time is the time taken for foods to exit the body following ingestion [69]. Whole gut transit time can be assessed using indigestible/radiopaque marker techniques, where the time from ingestion of the markers to the appearance in the feces is measured. Markers that are commonly used are radioactive or heavy metals, such as cobalt EDTA and chromium [70]. In a study conducted by Milne et al., it was reported that the mean whole gut transit time for 20 healthy horses on an alfalfa forage diet was about 29 h [71]. Pearson and Merritt also assessed the whole gut transit time of ponies on a hay diet using the same technique, and the mean retention time for chromium mordanted fiber and coEDTA-labelled liquid were reported to be 29.9 h and 31.3 h, respectively [72].

Several modifiable and non-modifiable factors can affect the GI passage rate of horses. These include breed, animal weight [73], level of exercise [59], and composition of feed [59,74].

Several studies have shown that light-weight horses have a faster mean whole gut transit time compared to heavy horses, thus, it can be concluded that mean retention time increases with increasing body weight [73]. However, there were also other studies in which the relationship between animal weight and mean retention time could not be established [75].

During exercise, there is a reduction in blood flow to the small intestine, this results in lower digestibility and an increase in passage rate. In an experiment conducted in horses by Weyenberg et al. [59], it was concluded that during exercise, there is longer retention times for the fluid phase marker and shorter retention time for the particle phase marker. This could be due to the increase in voluntary intake of feed or water, increasing gut motility. Hence, it is shown that exercise can have different effects on the retention time as digestibility is affected by the type of exercise as well as the amount of fluid and particles in the feed [59].

Feed particle size can also affect transit time. Weyenberg and colleagues also reported that transit time increases with decreasing feed particle size, and that passage rate increases with increasing bulk consumption of the long hay [59]. The passage rate is also affected by the water holding capacity of the feed. Feed consisting of hydrophilic polysaccharides can absorb water and hold it in the gut, hence, increasing transit time and retention time [59].

The composition of feed has also been shown to affect the gastrointestinal retention time, and the type of feed processing can also influence the digestibility of the feed, due to the gelatinization of starch and the denaturation of proteins [74]. For example, in a study carried out by Rosenfeld and Austbø [74], three (3) different types of grains–barley, maize, and wheat were used, and four different processes were performed on each type of grain, including ground, pelleted, extruded and micronized. It was found that maize had the longest retention time among the 3, and transit time was not significantly affected by the type of grain. It was also observed that pelleted grains had a longer transit time compared to ground grains, and their retention time was not significantly altered by the type of processing method. Thermal treatment can improve the digestibility of the starch and protein; thus, thermally treated grains tend to have longer transit and retention times. As transit time can be affected by particle size [59], grinding grains can minimize the differences in particle sizes, thereby, increasing feed utilization. However, it is noted that different experiments yield different results and the estimated retention times have large variations, indicating that external factors, such as the breed, and bodyweight of the horse can significantly affect their GIT transit time [74].

##### Orocecal Transit Time (OCTT)

Sutton et al. [76] utilized the lactose ^13^C-ureide breath test (LUBT), a safe and non-invasive way to evaluate the orocecal transit time in equines. The test was conducted using 3 different methods: an induced LUBT, non-induced LUBT, and a dual-isotope test [76,77] (Table 2). An induced LUBT was performed by administering a priming dose of ^12^C-LU to optimize equine lactose ^13^C-ureide (^13^C-LU) digestion [77]. A non-induced LUBT was conducted in the same fashion without the priming dose, and the dual-isotope test was always done following the same procedure but with an addition of ^13^C-octanoic acid. OCTT was evaluated by analyzing the ^13^C:^12^C ratio of breath samples using a mass spectrometer. Compared to non-induced LUBT test, the induced LUBT method has an excellent specificity due to sensitivity for lactose [76,77], however, the interpretation for the latter can be trickier due to the presence of by-products. The dual isotope test is also reliable method, but more complicated setup process is needed to make it operational.

### 3.2. Key Considerations for Oral Drug Delivery Development for Horses

The GIT of the horse and humans has many similarities, with differences between the two species lying at the organ level, rather than the molecular. The most obvious difference is the size of the gastrointestinal system. Humans have a significantly smaller large intestine compared to the large intestine of the horse which is multi-component and highly capacious [78]. There is also a major difference in the function of the cecum between humans and horses. For humans, the cecum’s main function is to absorb fluid and salts that remain after the completion of intestinal digestion [79]. While for horses, the cecum as part of a large intestine is a highly important site that provides an environment for the growth of microorganisms which favors fermentation, production of volatile fatty acids for energy, and synthesis of important vitamins. Fermentation products can change due to changes in the micro-environment of the bowel, such as changes in the pH of the large intestine as the gut microbiota can be affected [80]. Thus, there must be careful considerations when administering oral drugs that can cause significant changes to gut pH and GI microbiome in horses.

As mentioned before, the overall GIT function of the horse is like that of humans, thus, in some cases, oral formulations developed for humans with slight modifications can be bioequivalent when administered to horses. In a recent study [81], the use of human meloxicam tablets compounded into a form of molasses was investigated for improved palatability and ease of administration. It was found that essential pharmacokinetic parameters such as the area under the curve (AUC) and maximum concentration were within the defined limits of 80–125%, thus, this formulation was considered bioequivalent [81] to the reference tablet.

Horses can exhibit neophobia—a natural and genetic response that protects them against unknown poisonous substances [82]. However, this can be a barrier when oral drugs that are odorous need to be delivered, especially when voluntary ingestion is required. These medications have to be administered via nasogastric intubation, or mixed with feeds to facilitate voluntary ingestion [83]. There may be a need to use taste modifiers to improve the acceptability and palatability of odorous medications for horses. There can also be potential issues when administering the medication with food, for example, when administering tablets with a special coating such as enteric-coated (EC) tablets, the coating can be compromised through the mastication process. EC is to enable drugs to bypass the stomach (low pH) without disintegration; the EC then disintegrates and releases the active drug when pH is higher such as in the intestines. When the enteric coating is compromised for dosage forms delivering acid-labile drugs such as erythromycin, the bioavailability can significantly decrease [84]. Alternative dosage forms would have to be used instead or a different method of administration, to effectively deliver these kinds of medications. While administering the drug within the feed is a convenient method to deliver oral drugs, it might not always be the most appropriate method.

Compared to other species, the length and volume of the GIT in horses are much larger, approximately over 30 m and 150 L, respectively [85]. This can result in longer passage time and delay the absorption of orally delivered drugs. These may potentiate the need to modify dosage regimens in horses. For example, a single oral dose of anthelminthic drugs in horses such as albendazole is considered an effective treatment because of longer GI transit time, while smaller animals such as dogs with shorter GI transit time require multiple dosing [86]. The bioavailability of orally delivered drugs can also vary between individual horses [87]. Variation in oral bioavailability could be due to drug–drug interactions; for example, several studies demonstrated that pre-treatment of horses with ivermectin can decrease the oral bioavailability of fexofenadine. As when fexofenadine was administered IV with pre-treatment of ivermectin, no reduction in its AUC was observed [88]. The life-stage of the horse can also result in varied bioavailability. For example, oral absorption of amoxicillin in adult horses ranges from 2–10%; while in foals it is much higher, at 36–42% [1,89].

The pharmaceutical food effect is extensively researched in human pharmaceutics [90]; while much less is known in equines. It is, however, logical to apply this concept to veterinary pharmaceutics as well. The amount of feed and type of feed can potentially impact the absorption of oral drugs in horses, leading to erratic pharmacological responses. For example, in a fed state, the absorption of micro-encapsulated erythromycin was much lower when compared to the fasted state in horses [1,89]. More research must be conducted on the pharmaceutical food effect of oral dosage forms in horses to address this gap of knowledge. In Table 3, some information about the commercially available oral medicines for horses is listed.

### 3.3. Novel Oral Drug Delivery Systems and Technologies Developed for Horses

Doxycycline is an antibiotic that possesses many pharmaceutical advantages compared to its parent drug. However, a horse-customized formulation containing doxycycline is currently unavailable in the market. Zoyada et al. formulated 2 oral long-acting doxycycline hyclate formulations and evaluated their pharmacokinetics in horses [91]. It was concluded that the oral paste formulation can optimize the usage of doxycycline hyclate in horses, and enhance compliance due to ease of administration [91].

Enteric coating is a novel technology that is widely applied in human pharmaceutics [92]. Its purpose is to minimize the drug release in an acidic condition and to allow the release of the drug in an alkaline environment [92,93]. There have been several studies involved investigating the pharmacokinetics and pharmacodynamics of enteric-coated omeprazole in horses, and it was found that a dose of 1 mg/kg body weight (bwt) is effective in treating and preventing squamous gastric ulcers [94]. However, other studies show that the effect of enteric-coated omeprazole on its absorption is not significant, and thus, the effect of feeding on bioavailability may vary from different formulations. Due to the lack of available resources, further investigation in this area is required [93]. Birkmann [94] conducted a study evaluating the efficacy of two formulations, including the enteric-coated formulation and the powder paste formulation; and the results showed that the enteric-coated formulation was not significantly superior to the powder paste formulation. It is also worth noting that there was a 300% increase in AUC in fasted horses with the powder paste formulation, indicating that the timing of meals and omeprazole administration can affect drug absorption. GastroGard^®^ is a product containing omeprazole with an alkaline buffer used to protect the active drug from acid-mediated degradation [94]. The buffered paste can neutralize the acid in the stomach to prevent degradation of the drug. Despite being a useful formulation, this is a very costly product, and hence, it is not commonly used. However, there are other generic formulations containing omeprazole available in the market at a much lower price; despite results from some studies demonstrating that these generics are not as efficacious [95]. Plain omeprazole without acid protection is also available in the European market. However, there is no further study around these formulations. This gap in knowledge can open new doors to research into utilizing omeprazole through the art of equine drug formulation. Therefore, a further study with more focus on stable formulations of proton pump inhibitor (PPI) such as omeprazole will be useful.

Other NDDS that have potential to be administered orally to the horse include modified capsules, devices, or tablets utilizing osmosis for a prolonged release effect [96,97]. Principally, osmotic DDSs involves the expansion of the delivery system, followed by control expulsion of therapeutic agents in the GIT [96,97,98]. An example of such technology is the Elementary Osmotic Pump [98,99]. It consists of a therapeutic agent compressed into a tablet that is coated with a semi-permeable membrane; a designed orifice is generated in the membrane [98]. When the tablet is submerged within an aqueous medium, water permeates, and enters the tablet through osmosis and solubilizes the active agent [98]. As the semi-permeable membrane is non-extensive, the influx of water causes an increase in volume, resulting in an overall increase in hydrostatic pressure [98]. This pressure drives the saturated solution of the drug within the tablet out through the orifice, delivering the drug at a constant rate until the entire active agent is completely solubilized [98].

## 4. Topical and Intradermal Administration in Horse

### 4.1. Anatomy of Horse Skin

In mammalian species, the integumentary system plays an essential role in forming a physical barrier that ensures protection of homeostasis. Despite the high susceptibility to skin-related diseases experienced by domesticated animals, there is incomplete investigation of horse dermatology, and this appears to be an ongoing challenge for pharmaceutical development [100]. For that reason, the majority of the data and information related to equine skin features were extrapolated from other mammalian species, including humans, dogs, and cats [100].

**Figure 3:**
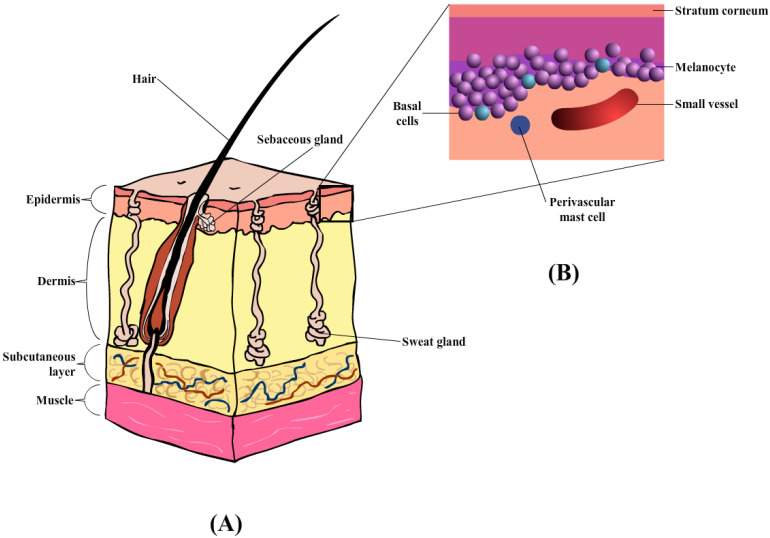
(**A**) Diagrammatic illustration of horse skin, containing three main layers: epidermis, dermis, and subcutaneous layers, together with hair follicles and sweat glands. (**B**) Enlarged illustration of the epidermis layer, containing the stratum corneum, melanocyte, basal, and perivascular mast cells.

The skin comprises the epidermis and dermis, which provides a physical barrier between the internal and external environment (Figure 3A) [101]. Similarly, the skin also plays an important role in the horse, specifically thermoregulation, and provides pigmentation. The outermost layer of the skin in the equine is the epidermis [100] containing multiple layers of cells, with an average thickness of about 0.0053 mm. Depending on the body region, the thickness of the epidermal layer is as thick as 6 mm, in areas such as the head, back, and rump [100]. The epidermis layer (Figure 3B) is composed of five layers of keratinocytes that undergoes constant proliferation, differentiation, and keratinization, contributing to the formation of the *stratum corneum* (SC) or the outermost layer. These three processes occur at a reasonable rate to maintain the mechanical barrier. According to Buechner-Maxwell and colleagues [100], the shedding cycle of a horse lasts for about 17 days, during which cell mitosis and superficial migration occur [100]. The keratinocytes are also surrounded by the intercellular lipid bilayers which helps prevent fluid loss upon any transdermal penetration [102]. Furthermore, the horse epidermis also consists of other cell types (or non-keratinocyte), namely Merkel cells, Langerhans’ cells, and Melanocytes. The roles of these specific cell types within the mammalian epidermis are generally the same across all species of mammals (e.g., Langerhans’ cells act as antigen-presenting cells that initiate cutaneous immune responses) [100].

Horses share similar histological arrangements with other mammals regarding the dermal layer. In the majority of mammalian species the dermal layer is divided into the superficial and deep dermis, which are highly vascularized with collagen-rich connective tissues, and intensive nerve supply [103]. The horse dermis also contains similar appendage features, such as hair follicles, sebaceous glands, sweat glands, and lymphatic vessels. Unlike other mammals, horses appear to have an extra layer that contributes to the increased thickness in the dorsal, croup, and back areas, where the thinnest (medial thigh and external genitalia) are on the ventral and medial surfaces of the limbs [100]. Due to the variations within the dermal layer the choice of injection site and needle placement should be carefully considered when administering injectables by the subcutaneous or intramuscular routes.

Moreover, the horse integumentary structure also contains the subcutis layer (also known as hypodermis or subcutaneous), primarily formed by adipocytes, loose fibroblasts, and collagen that aids in anchoring the dermis to the underlying tissues [102]. A typical structure of equine skin is illustrated in Figure 3 below [100].

### 4.2. The Key Considerations for Topical and Intradermal Drug Delivery Development for the Horse

The skin is the major protecting interface between the internal organs and the external environment; it is also considered the largest organ and is highly innervated. A high level of innervation allows horses to respond to external stimuli, demonstrating implications for their general well-being. Several similar obstacles are faced in the development of both human and equine formulations for topical/transdermal administration. While the basic skin anatomy is similar in all mammals; the thickness of the epidermis and dermis layer varies between species. Hence, the rate of percutaneous absorption is different between humans and horses, causing more challenges in developing topical products for systemic delivery in the horse [104].

Topically applied products come in different physical forms, including dusting powders (solid), creams ointments (semi-solids), and liquids (suspo-emulsion). One of the primary key concerns in equine topical/transdermal formulation development is the rate and extent to which active ingredients can penetrate into and through the skin [105]. This varies among many intended applications of topical formulations, such as (i) locally acting (corticosteroids and ectoparasiticides), (ii) systemic acting (oestradiol and testosterone patches); (iii) surface(sunscreens), or (iv) deep tissue targeting (NSAIDs) [105]. Hence, manufacturers must be aware of their intended indications for equine topical products, preparing an appropriate approach for a specific application.

Like the case of human products, for a systemic effect through topical formulation the drug needs to penetrate the SC or the major barrier of the skin [105,106]. Different regions of the equine body have different skin thicknesses, the density of hair follicles and glands, as well as vascularity and metabolic enzymes. In other words, differences in skin properties can result in regional differences in percutaneous drug penetration through equine skin [105]. Therefore, the site of application for topically applied formulation plays an important role, and they should always be carefully considered. For example, topical application of anti-inflammatory hydrocortisone and methylsalicylate on equine legs was found to have a quicker rate and higher level of absorption, compared to when it was administrated in other parts of the body [105].

Enhancing drug penetration through equine skin can be achieved using chemical and mechanical permeation enhancers. Chemical penetration accelerants interact with the lipid components of the skin, increasing its fluidity and causing the SC to swell [107,108]. These substances can also reduce the skin binding of drug molecules, hence, promoting their transportation through the skin. For a chemical to be considered a suitable penetration accelerant, the compound needs to be chemically stable; cutaneous waterproof, non-irritant, non-toxic, and compatible with other ingredients within the formulation [107]. Stah and Kietzmann [109] investigated the effects of six different permeation enhancers, as well as the effect of needle lengths on the delivery of transdermal lidocaine to equine skin. The study demonstrated the beneficial effects of equine skin micro-needle pretreatment, on topically applied lidocaine [109].

For effective delivery of topical products, adequate contact time between the formulation and equine skin is required [110]. To address issues with contact time, a few micro-vesicle formulations have been developed. For example, Novosomes *Vétoquinol* was fabricated into an extremely stable micro-vesicle, with the ability to resist hydrolysis caused by enzymatic activities, and with improved stability at higher temperatures up to 80 °C. Moreover, the negatively charged *Vétoquinol* product can form a stable attachment with the positively charged equine hairs, preventing the products from being removed due to rinsing or sweating [110]. *Spherulites^®^ is* an equine cleansing product that consists of 1 µm microparticles of plant-derived surfactants. This product contains chitosan and chitosanide which assist in forming a tightly bound film coating over the equine hair and skin. The tightly bound film increases the contact time of active ingredients with the skin, enhancing the topical drug delivery of active ingredients [110].

As many of the USA FDA-approved equine products are systemically absorbed, veterinarians need to consider systemic side effects when prescribing a topically applied product [111]. The systemic effect is a major challenge when obtaining the biowaiver/bioequivalent approval for topical products intended for localized effects [111]. Drug transportation across various epidermal layers is also affected by the skin absorption mechanism. This can be different between species; and within a species, chemical absorption from the skin could vary between seasons, and is also induced by the effects of sex hormones [104]. In other words, applying the percutaneous penetration data of one species to another without considering these interspecies variations is a risky and impractical approach. In addition to this, if the drug molecules from topical formulations are not well absorbed systemically, bioequivalence cannot be accessed from blood samples; instead, the in vivo efficacy and safety endpoints must be utilized. In this case, a higher number of test subjects need to be used to evaluate the dose–response relationship, instead of the typical dose–blood concentration relationship obtained from blood samples [112]. A number of these preparations available in the market are presented in Table 4 below.

### 4.3. Novel Topical and Intradermal Drug Delivery Systems and Technologies Development for Horses

One of the most common forms of skin neoplasia in the horse is the Equine sarcoid—Bovine papillomavirus (BPV) induced tumours commonly found in horses [113]. One of typical treatments for sarcoid involves the use of acyclovir topically [113], as it is easy for the owner to apply, and the drug is known to have minimal side effects. The topical application of acyclovir in humans is well understood, however, in horses it is not well studied and documented. Haspeslagh et al. [114] conducted a study to evaluate the transdermal delivery of acyclovir in both normal and sarcoid equine skin. The study revealed that drug penetration into the deeper dermal layers was significantly less in sarcoid skin, compared with normal skin [114].

Mills and Cross [115] conducted a study to evaluate the differences in drug penetration of fentanyl patches applied to different areas of equine skin. Skin samples were collected from the thorax, groin, and legs. It was demonstrated that drug penetration was better when the patch was applied to the thorax or the groin regions, compared to the leg. This could be due to the differences in cutaneous blood flow, appendageal density, and the thickness of the SC layer in the various regions of the skin [115].

In other studies, penetration enhancers (PEs) are included in topically applied formulations, to increase the drug permeation rate through the barrier membrane. This effect can be achieved via interactions with different components of the skin, as PEs are thought to increase fluidity in the intercellular lipid lamellae, as well as cause the SC to swell [109]. An example of a penetration enhancer is limonene, a substance that increases the percutaneous absorption of lipophilic and hydrophilic drugs. Ferrante et al. conducted a study evaluating the effects of 3 types of PEs–limonene, urea, and oleic acid, on diclofenac diethylamine (DD) permeation across equine skin [116]. Results obtained from this study revealed a significant increase in DD permeation, and all 3 types are equally useful in enhancing DD transdermal absorption.

However, it is challenging to develop ideal topical DDSs for horses. One of the main obstacles associated with the use of topical medications is the uncertainty of drug penetrability, which influences the degree/extent of the drug that entered the systemic circulation. Drug penetration is dependent on several factors, such as the extent of perfusion of the skin, and the number of hair follicles [117]. As a result, an important aspect to consider when applying topical formulation is the integrity of the horses’ SC layer. Pretreatments such as shaving, cleaning, or disinfection can irritate and damage the SC, which may cause changes to the drug efficacy, and systemic bioavailability and induce adverse effects. For example, Mills and Cross [102] conducted a study to demonstrate the effects skin pretreatment has on drug penetration. It was concluded that the destruction of the SC will reduce the skin’s barrier function, leading to an increase in systemic exposure [102]. Therefore, practitioners should be aware that the destruction of the SC layer increases systemic absorption, heightening equine pharmacological response, and increasing the risk of side effects.

Another potential issue is the extensive range of body sizes across and within equine species. Consequently, the ratio of the patch area to body weight restricts the number of drugs that can be delivered with this technology. As a result, equine patch development is not as feasible compared to patches devised for small animals like cats or dogs. However, patch development containing very potent drugs such as fentanyl, or other compounds where minimal exposure is required, might be possible for use in larger animals such as horses [118]. Therefore, the choice of incorporated drugs is important and should be considered during the development of equine patches.

## 5. Administration by Injection in the Horse

### 5.1. Anatomical Features

Intravenous administration can be applied to horses in various clinical situations, such as for anesthetic purposes before castration surgery, or for local analgesia in the treatment of acute laminitis [119]. Repeated treatments can sometimes be employed instead of a simple intravenous (IV) administration, such as in the case of lameness [120].

IV administration is often used with a catheter when there is a large volume of therapeutic compounds, electrolyte solutions or nutritional fluids to be delivered [120,121]. Catheter placement in this case can either be for short or long-term use, depending on the condition of the horses. Placement of the catheter is a complex process, with strict aseptic practice required, and thus, requires suitably trained personnel to perform the procedure [121,122]. Therefore, having a thorough anatomical understanding of the injection sites are very crucial.

**Figure 4:**
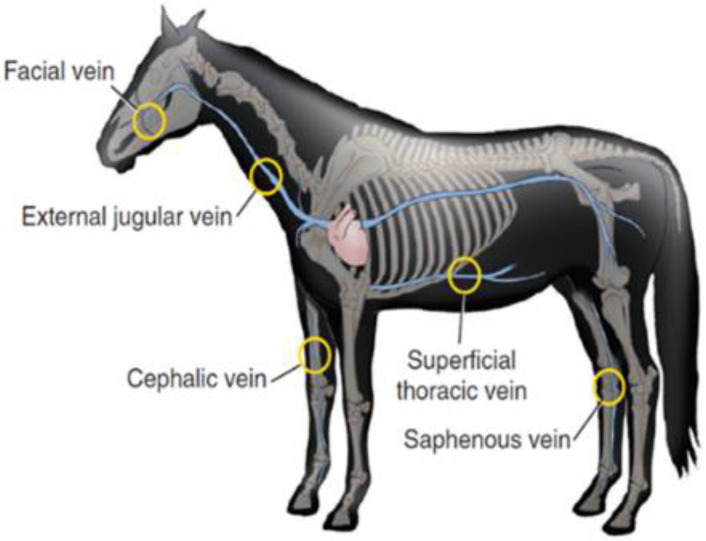
Anatomical sites for equine intravenous administration, including jugular, superficial thoracic, facial, cephalic, and saphenous veins. Reprint with permission from Orsini [124]. 2022, Elsevier.

Generally, the most common site of IV injection is the jugular vein, as demonstrated in Figure 4. Lorello and Orsini [121] reported that in emergency situation or under anaesthesia the cephalic vein and thoracic vein can also be used by most veterinarians, for the placement of various types of catheters with different lengths, diameters, and materials.

For medications that need to be administrated in large doses such and are deemed not suitable for IV route, it is often practical and convenient to administer them via the intramuscular (IM) route, into the large muscles actively used by the horse [123]. This enhances the drug absorption and reduces the risks of discomfort, such as pain and swelling at the site of injection. Moreover, the choice of muscles to inject must be carefully determined, allowing deep placements of needle tips without damaging adjacent ligaments, nerves, or blood vessels. Therefore, relatively safe injection sites with large muscle mass for IM route in horses include the rump are often chosen [123]. The other common anatomical site for equine intravenous administration is demonstrated in Figure 4.

The skin is another factor that can interfere with the action of injectable formulations, especially for intradermal and subcutaneous injections. The skin is the largest body organ in the horse, composing up to 12–24% of the total body weight, with thickness ranging from 1.47 to 4.57 mm [125,126]. The outer layer of the skin or the stratum corneum acts as a protective barrier against the movement of drug molecules across the skin into the body; hence, the disruption of the stratum corneum will thereby affect the barrier function of the skin [127]. The integrity of the SC in horses can be compromised before injections to improve drug penetration. This can be done simply by shaving the site of application, to eliminate the initial barrier [118,128,129]. Disinfecting the skin of horses with formulations containing alcohol before injecting could also help disrupt the stratum corneum [118,128,129].

### 5.2. The Key Considerations of the Injective Drug Delivery Development for Horse

When preparing immunogenic formulations, if horses are injected with sizable water-in-oil immunogens at one or two sites, there is a possibility that adverse local effects might occur [130]. Indeed, they might induce skin disruption, which might necessitate additional surgeries to remove the affected mass [130]. Therefore, the amount of administrated formulation is very crucial. For instance, emulsified immunogen preparation should be injected only in small volumes, such as 0.1–0.2 mL at multiple sites, reducing the risk of adverse local reactions at each site [130].

Various studies in cattle have demonstrated that decreasing the volume of active agents injected at a given IM site, by splitting the total volume and administering them into two separate sites, will significantly reduce muscle irritation and the extent of tissue damage [131]. This phenomenon can be attributed to the reduction of oil-based suspensions injected at the given location, consequently reducing local irritations [132]. Moreover, by spitting the dose, as well as distributing the total dose volume over more than one injection site, the absorption of the drug was delayed, prolonging the drug elimination process as compared to injecting a complete dose at one single site [132].

In addition to this, the ideal properties of injectable anesthetic formulations devised for the horses should have rapid onset of action, relatively short duration of action, the absence of biologically active metabolites, and a good quality of anesthesia recovery [133,134]. Furthermore, the total dose of epidural injections for local analgesia is determined by various factors, including the site of injection, the conformation of the animal skin, and the distance of the needle tip to the spinal cord of horses [135].

The type of surfactants or surface-active phospholipids (SAPLs) used as boundary lubricants in intra-articular administrations for horses should also be carefully considered. Two of the most commonly injected SAPLs in horses are phosphatidylcholine and hyaluronic acid (HA); which are well known for their anti-inflammatory and lubricative properties, without causing significant adverse effects when injected into a horse’s joints [136]. Interestingly, large quantities of prilocaine could be deposited within the horse joints with minimal synovitis when SAPLs are incorporated within propylene glycol (PG), a polymer commonly used in oral, topical, intravenous, and intramuscular formulations [136].

In addition to this, it is well established that various manufacturers have included preservatives in their equine formulations, to prevent possible bacterial and fungal contamination. Thus, it is essential to consider the effects that these chemicals might have on the physicochemical properties of active ingredients [137], eliminating the risk of inducing adverse reactions in horses. Most importantly, information regarding the safety profile and tolerability of the injected materials used to prepare injectable formulations must be thoroughly studied. Finally, the completed injections must not damage the structural components of horses’ organs, nor cause granulomatous synovitis and/or any other forms of overt inflammation [138,139,140].

With a continuously growing demand for injectable formulations for horses, manufacturers around the globe have invested intensively to produce safe and effective products. A number of these preparations available in the market are presented in the table below (Table 5).

### 5.3. Some Novel Injective Drug Delivery Systems and Technologies Development for Horses

Besides marketed products, the research efforts to fabricate novel injective formulations for horses continue to grow. For instance, a long-acting IM injection has recently been formulated to administer a once-per-week single dose of 2 g omeprazole (LA-OMEP), for the treatment of equine squamous and glandular gastric disease [141]. This non-irritant formulation successfully increased equine gastric pH by suppressing acid production. It was also found after the administration of LA-OMEP, gastric pH levels exceeded 4, for up to 7 days during the pilot pharmacodynamic studies, without dietary modification as required when oral omeprazole is used. Most importantly, this formulation has good stability at room temperature for extended durations, even though omeprazole is known to be extremely unstable.

In a study conducted by Rhodin and colleagues [138], a new sustained-release formulation of diclofenac (SYN321) was successfully developed for intra-articular administration to horses. Diclofenac release from SYN321 was quantifiable for approximately 4 days to at least 7 days, in plasma and urine, respectively. Clinical examination and objective lameness demonstrated the promising effect of SYN321 as a local joint NSAID treatment, without oblivious clinical evidence of side effects. Similarly, Petit et al. successfully fabricated an intra-articular and sustained release hydrogel composed of acetyl-capped poly(ε-caprolactone-*co*-lactide)-*b*-poly(ethylene glycol)-*b*-poly(ε-caprolactone-*co*-lactide) (PCLA-PEG-PCLA) copolymer loaded with celecoxib to inject into horse joints [138]. The extended-release profile of celecoxib for 4 weeks was also achieved [138]. Most importantly, there were no signs of damage in cartilage, demonstrating that these formulations were well tolerated and could be a promising approach for intra-articular drug delivery in horses [138]. In another study, single-dose long-acting polyethylene glycol (PEG) carrying oxytetracycline was developed into IV and IM formulations [142]. Pharmacokinetic results demonstrated that the long-acting oxytetracycline-PEG can be injected into horses at 20 mg/kg IM on a long-term basis, without altering microbial flora or inducing adverse gastrointestinal effects.

In the case of parenteral delivery, the drug is generally released continuously, however, in some scenarios, a pulsatile release is more effective. Such technology is commonly used to deliver substances such as estradiol and corticosteroids. Moreover, implantable devices are easily inserted and are less likely to cause tissue irritation at the site of administration. For example, the Alzet osmotic pump is an implantable delivery system that allows drug release to be programmed to follow zero-order kinetics or pulsatile dose release at fixed time intervals [143]. Thrombophlebitis or local cellulitis is inevitable in long-term venous access, regardless of the exact form used, including catheterization or intramuscular. In addition to this, there is difficulty in choosing an appropriate-sized needle/catheter [12,121]. This is an area where implants provide an advantage over catheterization.

Rezende et al. conducted a study to investigate the possibility of propofol application as a primary anesthetic for extended periods [144]. In this study, 5% micellar microemulsion propofol formulation was given to all subjects on two occasions: starting with a single bolus followed by a 3 h continuous infusion (phase II) [145]. During anesthesia and recovery, the researchers monitored cardiovascular, respiratory, and biochemical parameters [144]. Based on the anesthetic induction and recovery characteristics, the microemulsion propofol formulation demonstrated similar results compared to commercially available propofol preparation, after being administered as a continuous infusion for 3 h [145]. However, further studies are required to evaluate the limits of safety and clinical applicability of these formulations [144].

## 6. Challenges in NDDS Development for the Horse

Dosage forms for horses are essentially like those used in humans; yet in some cases, the unique biological characteristics of horses might cause different outcomes. Controlled release drug delivery technology in horses provides many advantages, such as minimizing animal handling, reducing stress to horses and owners and carers, as well as reducing the cost of treatment. However, despite these advantages, there are relatively few products that successfully reach the marketing stage. Indeed, Rathbone and Brayden attributed this obstacle to the complexity of the environments animals are exposed to, resulting in undesired and unexpected effects on stability, release characteristics as well as the physical and chemical behaviors of drug substances [144].

One of the main challenges faced by a veterinary pharmaceutical company is the lack of financial incentives. Generally, the final sales of a product represent its success; therefore, product development must be tailored accordingly, to achieve an effective product at an affordable cost. In addition to this, the weight of an animal will change throughout its life; hence, different strengths and volumes of drugs must be carefully catered to meet these variations.

Finally, the lack of literature precedence and expertise in this specific field of pharmaceutics remains a primary obstacle during the development of veterinary products. Even with a cross-over knowledge in the development of human products and veterinary products, the research specifically into veterinary NDDs is still in its infancy. Therefore, expending the pool of knowledge through innovative research in this field will be highly beneficial. However, this is not always easy, as the pharmaceutical specialists and tools to achieve this goal are still lacking [144].

## 7. Conclusions and Authors’ Insight into the Future Trends of Veterinary Formulations

Extensively researched areas in veterinary drug delivery mainly revolved around convenient dosing, long-acting formulations, and needle-less injections [146]. As a result, convenience has always been a top priority in veterinary formulations, ensuring the ease of dosing and administration. Less frequent dosing, e.g., once-a-day, is usually considered ideal. However, due to variations in gastric retention and intestinal transit time among animal species, the sustained-release oral formulations for once-a-day may not always be feasible. Topical formulations become a popular option for convenient administration; however, the efficacy of drug delivery is highly dependent on the pharmacokinetics and pharmacodynamics profiles, and environment impacts of washed off antiparasitic formulations if becoming a key concern. Taking all this information into account, an opportunity for veterinary NDDSs arises through the enhancement of current delivery platforms, allowing less frequent dosing formulations to be developed [146].

There is currently an increasing interest in developing NDDSs that can minimize tissue residues and lower the risk of local irritation. However, delivering such technologies to animals can be challenging, and they often require specialized equipment. To address this issue, novel polymers and therapeutic molecules can be modified in a fashion that enables their administration by conventional syringes [146]. This will greatly reduce the risks and costs of specialized equipment and experts needed.

In addition to this, the use of needles in animals has always been unfavorable, due to the risk of injury and unnecessary stress put upon the treated animal. Needleless injectors are gaining global attention for use in humans, some of which have obtained regulatory approvals. In addition to this, the use of needleless injections in animals is very complex, due to the variations in skin thickness regionally and changes with age.

One example of the cutting-edged delivery systems is *Aservo EquiHaler*–the first intranasal product developed to deliver ciclesonide-an anti-inflammatory medicine [147]. The product demonstrated promising outcomes in reducing the severity of asthma and it is very well-tolerated in equines. Despite the initial success and a growing interest in this route of delivery systems, there is still very limited evidence for the wider application of such technologies in equines. In spite of these obstacles, it is very likely more novel delivery methods like this will be commercialized in the coming years, enhancing the compatibility and efficacy in equines health.

## Figures and Tables

**Table 1:** Key differences between foals and adult horses.

Factor	Observation	Discussion	Ref.
Body Weight	Foals can grow rapidly. Weight gain ≈ 1.15 ± 0.17 kg/day; however, bodyweight gain is dependent on birth weight and breed.	Constant need to adjust doses of medications that are dosed according to weight. This is especially important for drugs with a low therapeutic index.	[35,36]
Oral Bioavailability	The bioavailability of oral drugs is different in foals and mature horses. Antibiotics such as ampicillin are poorly absorbed in adult horses compared to foals.	Gastrointestinal absorption, and/or binding to feed might induce the difference in oral bioavailability	[36,37,38]
Volume of Distribution	Foals have a significantly higher percentage of water to fat ratio, compared to adult horses.	Foals may require higher doses of hydrophilic drugs such as aminoglycosides, due to a larger apparent volume of distribution in foals.	[36,39,40]
Serum Protein Concentration	Foals have lower serum protein concentration compared to adult horses.	Important for highly protein-bound drugs. Drug molecules that remain bound to serum protein are therapeutically inactive, hence differences in serum protein should be considered and adjustments to the medication dose should be made if needed.	[36,41]
Metabolism & Excretion	Glomerular filtration rate and plasma flow rate of foals can reach adult values within 1–2 days of age.	There is a lack of information on time taken for a foal’s kidney to achieve maturity of transported mediated tubular excretion, which is important for renally cleared drugs.	[36,42]
	Foals have underdeveloped hepatic microsomal enzyme pathways, compared to adult horses.	This can lead to prolong half-life for medications that are primarily metabolized by the liver. The doses of drugs that are primarily cleared by the liver need to be modified, to achieve safe and effective therapy in foals.	
Urine	The urine of an adult horse is more alkaline.	Thus, acidic drugs are more likely to be reabsorbed in foals; while basic drugs are more likely to be absorbed in adult horses.	[36]

**Table 2:** The OCTT value measurement.

Method	OCTT (h)	Method of OCTT Measurement	Ref.
1	3.24 ± 0.65	Induced LUBT	[76]
2	5.12 ± 1.01	Non-induced LUBT	
3	3.66 ± 0.6	Dual isotope test	

**Table 3:** Examples of Marketed/Registered equine oral products and their indications.

Product Name	Active Constituent	Indication	Holder/Manufacture	Registration Year
Sedalin oral gel	Acepromazine	Sedative agent in horses Relieve pain and distress	Vetoquinol Limited (UK)	1996
Griseofulvin powder (microsized)	Griseofulvin	Treatment for ringworm	Bimeda Inc. (USA)	2014
GastroGard^®^	Omeprazole	Inhibition of gastric acid secretion. Healing gastric ulcers and preventing their recurrence.	AstraZeneca Pty Ltd. (UK)	2005
Pergoscend film-coated tablets	Pergolide	Equine Cushing’s Disease	Dechra Veterinary Products (UK)	2021
EQUIOXX^®^ tablets	Firocoxib	Control of pain and inflammation. First and only approved NSAID for horses.	AUDEVARD (UK)	2017
Resvantage Equine^®^	Resveratrol	Promote overall wellbeing of horses Increase energy and endurance Enhance anti-inflammation activity	Advantage Biosciences Inc. (USA)	N/A
Gastropell Forte Oral Paste	Omeprazole	Treatment and and prevention of gastric ulcers in racehorses	RANDLAB Pty Ltd. (Australia)	2013
PULSE-8 liquid vitamin supplement	Vitamins	Enhanced utilisation of high energy diets Performance enhancement in racehorces	VIRBAC (Australia) Pty Ltd. (Australia)	2020
UlcerGard^®^ (for horses)	Omeprazole	Inhibition of gastric acid secretion. Healing gastric ulcers and preventing their recurrence.	Merial Ltd. (USA)	2004

**Table 4:** Examples of Marketed/Registered equine topical/transdermal products and their indications.

Product name	Active Constituent	Indication	Manufacture	Registration Year
ExCellR8TM	Proteins Cytokines Anti-Inflammatory molecules	Protein cream for legs and joints Anti-Inflammatory caused by injuries to muscles, joints, tendons, and ligaments	Equi-Stem Biotechnologies LLC (USA)	2016
Laminil© cream	Cromolyn Nedocromil	Treatment for early laminitis, inflammation, and retarded hoof growth	Willowcroft Pharm Inc. (USA)	2009
Surpass^®^ cream	Diclofenac Sodium	Control of osteoarthritis-induced pain and inflammation.	Boehringer Ingelheim Vetmedica, Inc. (USA)	2004
Fungazol Cream^®^	Econozole Nitrate	Anti-fungal	Ranvet Pty Ltd. (Australia)	2016
Equiwinner™	Electrolytes	Elimination of bleeding (EIPH), non-sweating (anhidrosis) headshaking.	Signal Health group (USA)	2003

**Table 5:** Examples of Marketed/Registered equine injectable products and their indications.

Product Name	Active Constituent	Indication	Manufacture	Registration Year
NV Glucosamine 200 Injection	Acetyl-d-Glucosamine	Anti-Inflammatory, Anti-Arthritic, and chondroprotective Agent	Ceva Animal Health Pty Ltd. (Australia)	March 2013
NV Pentosan Equine Injection	Pentosan Polysulfate Sodium	Anti-inflammatoryjointand stimulant of cartilage synthesis	Ceva Animal Health Pty Ltd. (Australia)	March 2016
Arti-Cell Forte Suspension for Injection	Equine peripheral blood-derived mesenchymal stem cells	Reduction of recurrent lameness associated with joint inflammation	Boehringer Ingelheim Vetmedica GmbH (UK)	March 2019
HY-50 VET	Hyaluronate Sodium	Intra-articular and IV treatment of joint dysfunction induced lameness	Dechra Limited (UK)	January 2016
Batphol^®^	Vitamin B complex Choline	Vitamin B complex and choline supplements	Ranvet Pty Ltd. (Australia)	July 2017
Recocam	Meloxicam	Treatment of musculoskeletal disorders	Bimeda Pty Ltd. (Australia)	August 2015
Banamine^®^	Flunixin Meglumine	Injectable nonsteroidal anti-inflammatory	Merck & Co. Inc. (USA)	July 2011
Trivetrin^®^	Trimethoprim Sulfadoxine	Antibacterial (slow IV injection)	Vet Pharma Friesoythe GmbH (Germany)	N/A

## Data Availability

Not applicable.

## References

[1] Papich M.G. (2001). Current concepts in antimicrobial therapy for horses. Proc. Annu. Conv. AAEP.

[2] Mitchell C., Fugler L.A., Eades S. (2014). The management of equine acute laminitis. Vet. Med. Res. Rep..

[3] Kauter A., Epping L., Semmler T., Antao E.-M., Kannapin D., Stoeckle S.D., Gehlen H., Lübke-Becker A., Günther S., Wieler L.H. (2019). The gut microbiome of horses: Current research on equine enteral microbiota and future perspectives. Anim. Microbiome.

[4] Schnepf A., Bienert-Zeit A., Ertugrul H., Wagels R., Werner N., Hartmann M., Feige K., Kreienbrock L. (2020). Antimicrobial Usage in Horses: The Use of Electronic Data, Data Curation, and First Results. Front. Vet. Sci..

[5] Maxwell L., Cole C., Bentz B., Maxwell L. (2015). Horse of a different color: Peculiarities of equine pharmacology. Equine Pharmacology.

[6] Knottenbelt D.C., Malalana F., Knottenbelt D.C., Malalana F. (2015). Part 3—Index of Drugs Used in Equine Medicine. Saunders Equine Formulary.

[7] Talevi A. (2021). Oral Drug Delivery. The ADME Encyclopedia: A Comprehensive Guide on Biopharmacy and Pharmacokinetics.

[8] Carr E.A., Geor R.J., Harris P.A., Coenen M. (2013). 41—Assisted enteral and parenteral feeding. Equine Applied and Clinical Nutrition.

[9] Horspool Q.A., Horspool L.J.I. (1995). Stability of penicillin G, ampicillin, amikacin and oxytetracycline and their interactions with food in in vitro simulated equine gastrointestinal contents. Res. Vet. Sci..

[10] Rosa B. (2020). Equine Drug Transporters: A Mini-Review and Veterinary Perspective. Pharmaceutics.

[11] Davis M., Williams R., Chakraborty J., English J., Marks V., Ideo G., Tempini S. (1978). Prednisone or prednisolone for the treatment of chronic active hepatitis? A comparison of plasma availability. Br. J. Clin. Pharmacol..

[12] Crabtree N.E., Epstein K.L. (2021). Current Concepts in Fluid Therapy in Horses. Front. Vet. Sci..

[13] González-Medina S., Nout-Lomas Y.S., Landolt G. (2022). Unintentional intracarotid injections in the horse—15 cases (2010–2020). Equine Vet. Educ..

[14] Saxen M.A., Dean J.A. (2016). Chapter 17—Pharmacologic Management of Patient Behavior. McDonald and Avery’s Dentistry for the Child and Adolescent.

[15] Ibeanu N., Egbu R., Onyekuru L., Javaheri H., Khaw P.T., Williams G.R., Brocchini S., Awwad S. (2020). Injectables and Depots to Prolong Drug Action of Proteins and Peptides. Pharmaceutics.

[16] Rahnfeld L., Luciani P. (2020). Injectable Lipid-Based Depot Formulations: Where Do We Stand?. Pharmaceutics.

[17] Shaw H. (2015). Intramuscular injection. Nurs. Stand..

[18] Firth E.C., Nouws J.F., Driessens F., Schmaetz P., Peperkamp K., Klein W.R. (1986). Effect of the injection site on the pharmacokinetics of procaine penicillin G in horses. Am. J. Veter-Res..

[19] Wagner B.K., Nixon E., Robles I., Baynes R.E., Coetzee J.F., Pairis-Garcia M.D. (2021). Non-Steroidal Anti-Inflammatory Drugs: Pharmacokinetics and Mitigation of Procedural-Pain in Cattle. Animals.

[20] Peek S.F., Semrad S.D., Perkins G.A. (2003). Clostridial myonecrosis in horses (37 cases 1985–2000). Equine Vet. J..

[21] Stewart S., Richardson D.W., Auer J.A., Stick J.A., Kümmerle J.M., Prange T. (2019). Chapter 7—Surgical Site Infection and the Use of Antimicrobials. Equine Surgery.

[22] Greenwood F.S.A.D. (1979). Action and interaction of penicillin and gentamicin on enterococci. J. Clin. Pathol..

[23] B I Asmar M.P., Dajani A.S. (1988). Antagonistic effect of chloramphenicol in combination with cefotaxime or ceftriaxone. Antimicrob. Agents Chemother..

[24] Wójcikowski J., Danek P.J., Basińska-Ziobroń A., Pukło R., Daniel W.A. (2020). In vitro inhibition of human cytochrome P450 enzymes by the novel atypical antipsychotic drug asenapine: A prediction of possible drug–drug interactions. Pharmacol. Rep..

[25] Isoherranen N., Foti R.S., Ma S., Chowdhury S.K. (2020). Chapter 9—In vitro characterization and in vitro to in vivo predictions of drug-drug interactions. Identification and Quantification of Drugs, Metabolites, Drug Metabolizing Enzymes, and Transporters.

[26] Barry M., Feely J. (1990). Enzyme induction and inhibition. Pharmacol. Ther..

[27] Hakkola J., Hukkanen J., Turpeinen M., Pelkonen O. (2020). Inhibition and induction of CYP enzymes in humans: An update. Arch. Toxicol..

[28] Tornio A., Filppula A.M., Niemi M., Backman J.T. (2019). Clinical Studies on Drug–Drug Interactions Involving Metabolism and Transport: Methodology, Pitfalls, and Interpretation. Clin. Pharmacol. Ther..

[29] Li H., Sheng Y., Li W., Yuan L. (2022). Recent Advances in Molecular Fluorescent Probes for CYP450 Sensing and Imaging. Chemosensors.

[30] Zhao M., Ma J., Li M., Zhang Y., Jiang B., Zhao X., Huai C., Shen L., Zhang N., He L. (2021). Cytochrome P450 Enzymes and Drug Metabolism in Humans. Int. J. Mol. Sci..

[31] Hammer H., Schmidt F., Marx-Stoelting P., Pötz O., Braeuning A. (2021). Cross-species analysis of hepatic cytochrome P450 and transport protein expression. Arch. Toxicol..

[32] Loureiro A.I., Rebouta J., Bonifacio M.-J., Soares-da-Silva P. (2020). In vitro Species Different Metabolism and CYP Phenotyping of Zamicastast. FASEB J..

[33] Vimercati S., Elli S., Jagannathan V., Pandey A.V., Peduto N., Leeb T., Mevissen M. (2019). In silico and in vitro analysis of genetic variants of the equine CYP3A94, CYP3A95 and CYP3A97 isoenzymes. Toxicol. Vitr..

[34] Esteves F., Rueff J., Kranendonk M. (2021). The Central Role of Cytochrome P450 in Xenobiotic Metabolism—A Brief Review on a Fascinating Enzyme Family. J. Xenobiot..

[35] Heidler B., Aurich J.E., Pohl W., Aurich C. (2004). Body weight of mares and foals, estrous cycles and plasma glucose concentration in lactating and non-lactating Lipizzaner mares. Theriogenology.

[36] Magdesian K.G., Cole C., Bentz B., Maxwell L. (2015). Foals are not mini horses. Equine Pharmacology.

[37] Furr M.O., Mogg T. (2003). Antimicrobial Treatment of Neonatal Foals. Vet Pract. Mag..

[38] Ensink J.M., Moi A., Vulto A.G., Tukker J.J. (2011). Bioavailability of pivampicillin and ampicillin trihydrate administered as an oral paste in horses. Vet. Q..

[39] Cymbaluk N.W., Geor R., Harris P.A., Coenen M. (2013). Equine Applied and Clinical Nutrition.

[40] Baggot J.D. (1994). Drug therapy in the neonatal foal. Vet. Clin. N. Am. Equine Pract..

[41] Gonzalez D., Schmidt S., Derendorf H. (2013). Importance of Relating Efficacy Measures to Unbound Drug Concentrations for Anti-Infective Agents. Clin. Microbiol. Rev..

[42] Lippi I., Bonelli F., Meucci V., Vitale V., Sgorbini M. (2019). Estimation of glomerular filtration rate by plasma clearance of iohexol in healthy horses of various ages. J. Vet. Intern. Med..

[43] Frape D. (2008). Equine Nutrition and Feeding.

[44] Joyner M.J., Casey D.P. (2015). Regulation of increased blood flow (hyperemia) to muscles during exercise: A hierarchy of competing physiological needs. Physiol. Rev..

[45] Sohail M.U., Yassine H.M., Sohail A., Thani A.A.A. (2019). Impact of Physical Exercise on Gut Microbiome, Inflammation, and the Pathobiology of Metabolic Disorders. Rev. Diabet. Stud..

[46] Lorenzo-Figueras M., Merritt A.M., Hinchcliff K.W., Kaneps A.J., Geor R.J. (2014). 46—Effects of exercise on gastrointestinal function. Equine Sports Medicine and Surgery.

[47] Sutton D.G.M. (2003). Stable Isotope Tracer Studies for the Measurement of Equine Gastrointestinal Motility.

[48] Van Weyenberg S., Sales J., Janssens G.P.J. (2006). Passage rate of digesta through the equine gastrointestinal tract: A review. Livest. Prod. Sci.-Livest. Prod. Sci..

[49] Merritt A.M., Julllian V. (2013). Gastrointestinal physiology. Equine Applied and Clinical Nutrition.

[50] Hepburn R. (2011). Gastric ulceration in horses. Practice.

[51] Easley J. Introduction to Digestive Disorders of Horses. Merck Sharp & Dohme Corp. (MSD) Manual. https://www.msdvetmanual.com/horse-owners/digestive-disorders-of-horses/introduction-to-digestive-disorders-of-horses.

[52] Sufit E., Houpt K.A., Sweeting M. (1985). Physiological stimuli of thirst and drinking patterns in ponies. Equine Vet. J..

[53] Argenzio R.A., Lowe J.E., Pickard D.W., Stevens C.E. (1974). Digesta passage and water exchange in the equine large intestine. Am. J. Physiol..

[54] Freeman D.E. (2021). Effect of Feed Intake on Water Consumption in Horses: Relevance to Maintenance Fluid Therapy. Front. Vet. Sci..

[55] Martinsen T.C., Fossmark R., Waldum H.L. (2019). The Phylogeny and Biological Function of Gastric Juice-Microbiological Consequences of Removing Gastric Acid. Int. J. Mol. Sci..

[56] Murray M.J., Schusser G.F. (1993). Measurement of 24-h gastric pH using an indwelling pH electrode in horses unfed, fed and treated with ranitidine. Equine Vet. J..

[57] Murray M.J., Grodinsky C. (1989). Regional gastric pH measurement in horses and foals. Equine Vet. J..

[58] Cho E.-A., Kim M.S., Cha Y.B., Lee M.-S., Song T. (2019). Evaluation of Gastric Emptying Time of a Rice-Based Meal Using Serial Sonography. BioMed Res. Int..

[59] Bennett P., Oza U.D., Trout A.T., Mintz A. (2016). Chapter 70Gastric Emptying. Diagnostic Imaging: Nuclear Medicine.

[60] Jacoby H.I. (2017). Gastric Emptying. Reference Module in Biomedical Sciences.

[61] Farrell M.B. (2019). Gastric Emptying Scintigraphy. J. Nucl. Med. Technol..

[62] Bahr A., O’Conor M., Roussel A.J., Cohen N.D. Evaluation of solid-phase gastric emptying in horses with induced gastric ulcers. Proceedings of the 7th Equine Colic Research Symposium.

[63] Levy M., Sojka J. (1991). Control of gastric emptying in the horse: Effect of cisapride (abstract). Proceedings of the 4th Equine Colic Research Symposium.

[64] Wuestenberghs F., Juge M., Melchior C., Desprez C., Leroi A.-M., Gourcerol G. (2019). Association Between Symptoms, Quality of Life, and Gastric Emptying in Dyspeptic Patients. J. Neurogastroenterol. Motil..

[65] Bharucha A.E., Camilleri M., Veil E., Burton D., Zinsmeister A.R. (2013). Comprehensive assessment of gastric emptying with a stable isotope breath test. Neurogastroenterol. Motil..

[66] Métayer N., Lhôte M., Bahr A., Cohen N.D., Kim I., Roussel A.J., Julliand V. (2004). Meal size and starch content affect gastric emptying in horses. Equine Vet. J..

[67] Asnicar F., Leeming E.R., Dimidi E., Mazidi M., Franks P.W., Al Khatib H., Valdes A.M., Davies R., Bakker E., Francis L. (2021). Blue poo: Impact of gut transit time on the gut microbiome using a novel marker. Gut.

[68] Müller M., Canfora E.E., Blaak E.E. (2018). Gastrointestinal Transit Time, Glucose Homeostasis and Metabolic Health: Modulation by Dietary Fibers. Nutrients.

[69] Lee Y.Y., Erdogan A., Rao S.S. (2014). How to assess regional and whole gut transit time with wireless motility capsule. J. Neurogastroenterol. Motil..

[70] Hall M.B., Van Soest P.J. (2019). Stability of the liquid markers chromium (III) and cobalt (III)-EDTA in autoclaved, clarified rumen fluid. J Dairy Sci..

[71] Milne E.M., Doxey D.L., Woodman M.P., Cuddeford D., Pearson P.A. (1996). An evaluation of the use of cisapride in horses with chronic grass sickness (equine dysautonomia). Br. Vet. J..

[72] Pearson R.A., Merritt J.B. (1991). Intake, digestion and gastrointestinal transit time in resting donkeys and ponies and exercised donkeys given ad libiitum hay and straw diets. Equine Vet. J..

[73] Miraglia N., Poncet C., Rosset W.M. (1992). Effect of feeding level, physiological state and breed on the rate of passage of particulate matter through the gastrointestinal tract of the horse. Ann. Zootech..

[74] Rosenfeld I., Austbø D. (2009). Effect of type of grain and feed processing on gastrointestinal retention times in horses. Am. Soc. Anim. Sci..

[75] Uden P., Rounsaville T.R., Wiggans G.R., Soest P.J.V. (1982). The measurement of liquid and solid digesta retention in ruminants, equines and rabbits given timothy (Phleum pratense) hay. Br. J. Nutr..

[76] Sutton D.G.M., Preston T., Love S. (2011). Application of the lactose 13C-ureide breath test for measurement of equine orocaecal transit time. Equine Vet. J..

[77] Sutton D.G.M., Preston T., Love S. (2011). In vitro validation of the lactose 13C-ureide breath test for equine orocaecal transit time measurement. Equine Vet. J..

[78] Furness J.B., Cottrell J.J., Bravo D.M. (2015). Comparative gut physiology symposium: Comparative physiology of digestion. J. Anim. Sci..

[79] Britannica E. (2018). Cecum Anatomy. Encyclopaedia Britannica.

[80] Johnson M.A., Anthony T., Blikslager N.A.W., Tim S., Mair J., Moore N. (2017). The Equine Acute Abdomen.

[81] Vivancos M., Barker J., Engbers S., Fischer C., Frederick J., Friedt H., Cribb A.E. (2015). Pharmacokinetics and bioequivalence of 2 meloxicam oral dosage formulations in healthy adult horses. Can. Vet. J..

[82] Lee K.E., Kim J.G., Lee H., Kim B.S. (2021). Behavioral and cardiac responses in mature horses exposed to a novel object. J. Anim. Sci. Technol..

[83] Brayden D.J., Cunningham F. (2010). Comparative and Veterinary Pharmacology. Handbook of Experimental Pharmacology.

[84] Maxwell L., Cole C., Bentz B., Maxwell L. (2015). Section 1 General Review Section—Horse of a different color: Peculiarities of Equine Pharmacology 3. Equine Pharmacology.

[85] Ericsson A.C., Johnson P.J., Lopes M.A., Perry S.C., Lanter H.R. (2016). A Microbiological Map of the Healthy Equine Gastrointestinal Tract. PLoS ONE.

[86] Corry A. New Drug-Albendazole. https://bovavet.com.au/blog/post.php?entry=18.

[87] Jersele A., Smiljanic T. (2012). Comparative Veterinary Pharmacokinetics. Readings in Advance Pharmacokinetics—Theory, Methods and Applications.

[88] Olsén L. (2007). Drugs in Horses: Pharmacokinetics and Pharmacodynamics.

[89] Giguère S., Cohen N., Keith Chaffin M., Slovis N., Hondalus M., Hines S., Prescott J. (2011). Diagnosis, Treatment, Control, and Prevention of Infections Caused by Rhodococcus equi in Foals. J. Vet. Intern. Med..

[90] Welling P.G. (1996). Effects of food on drug absorption. Annu. Rev..

[91] Zozaya H., Gutierrez L., Bernad M.J., Sumano H. (2013). Pharmacokinetics of a peroral single dose of two long-acting formulations and an aqueous formulation of doxycycline hyclate in horses. Acta Vet. Scand..

[92] Porter S.C., Adejare A. (2021). Chapter 27—Coating of pharmaceutical dosage forms. Remington.

[93] Sykes B.W., Underwood C., Mcgowan C.M., Mills P.C. (2015). Pharmacokinetics of intravenous, plain oral and enteric-coated oral omeprazole in the horse. J. Vet. Pharmacol. Ther..

[94] Birkmann K., Junge H.K., Maischberger E., Eser M.W., Schwarzwald C.C. (2014). Efficacy of Omeprazole Powder Paste of Enteric-Coated Formulation in Healing of Gastric Ulcers in Horses. J. Vet. Intern. Med.

[95] Merritt A.M., Sanchez L.C., Burrow J.A., Church M., Ludzia S. (2003). Effect of GastroGard and three compounded oral omeprazole preparations on 24h intragastric pH in gastrically cannulated mature horses. Equine Vet. J..

[96] Wong P.L.-S., Theeuwes F., Larsen S.D., Dong L.C. (1996). Osmotic Device for Delayed Delivery of Agent. US Patent.

[97] Ogueri K.S., Shamblin S.L. (2022). Osmotic-controlled release oral tablets: Technology and functional insights. Trends Biotechnol..

[98] Verma R.K., Mishra B., Garg S. (2000). Osmotically controlled oral drug delivery. Drug Dev. Ind. Pharm..

[99] Shokri J., Ahmadi P., Rashidi P., Shahsavari M., Rajabi-Siahboomi A., Nokhodchi A. (2008). Swellable elementary osmotic pump (SEOP): An effective device for delivery of poorly water-soluble drugs. Eur. J. Pharm. Biopharm..

[100] Buechner-Maxwell T.M.V., Wong D. (2005). Equine Skin: Structure, Immunologic Function, and Methods of Diagnosing Disease. Compendium.

[101] Someya T., Amagai M. (2019). Toward a new generation of smart skins. Nat. Biotechnol..

[102] Mills P.C., Cross S.E. (2006). The effects of equine skin preparation on transdermal drug penetration in vitro. Can. J. Vet. Res..

[103] Sloet van Oldruitenborgh-Oosterbaan M.M., Grinwis G.C.M. (2016). Basics of equine dermatology. Equine Vet. Educ..

[104] Monteiro-Riviere N.A., Bristol D.G., Manning T.O., Rogers R.A., Riviere J.E. (1990). Interspecies and interregional analysis of the comparative histologic thickness and laser Doppler blood flow measurements at five cutaneous sites in nine species. J. Investig. Dermatol..

[105] Mills P., Cross S. (2007). Penetration of Pharmacological Agents through Equine Skin. Rural Industries Research and Development Corporation Report.

[106] Lampe M.A., Burlingame A.L., Whitney J., Williams M.L., Brown B.E., Roitman E., Elias P.M. (1983). Human stratum corneum lipids: Characterization and regional variations. J. Lipid Res..

[107] Magnusson B., Walters K., Roberts M. (2001). Veterinary drug delivery: Potential for skin penetration enhancement. Adv. Drug Deliv. Rev..

[108] Dayan N., Rosen M.R. (2005). 4—Delivery System Design in Topically Applied Formulations: An Overview. Delivery System Handbook for Personal Care and Cosmetic Products.

[109] Stahl J., Kietzmann M. (2014). The effects of chemical and physical penetration enhancers on the percutaneous permeation of lidocaine through equine skin. BMC Vet. Res..

[110] Bloom P. (2009). Topical Treatments for Skin Diseases.

[111] Baynes R.E., Riviere J., Franz T., Monteiro-Riviere N.A., Lehman P., Peyrou M., Toutain P.L. (2012). Challenges obtaining a biowaiver for topical veterinary dosage forms. J. Vet. Pharmacol..

[112] Martinez M., Langston C., Martin T., Conner D. (2002). Challenges associated with the evalution of veterinary product bioequivalence: An AAVPT perspective. J. Vet. Pharmacol..

[113] Haspeslagh M., Jordana Garcia M., Vlaminck L.E.M., Martens A.M. (2017). Topical use of 5% acyclovir cream for the treatment of occult and verrucous equine sarcoids: A double-blinded placebo-controlled study. BMC Vet. Res..

[114] Haspeslagh M., Taevernier L., Maes A.A., Vlaminck L.E.M., Spiegeleer B.D., Croubels S.M., Martens A.M. (2016). Topical distribution of acyclovir in normal equine skin and equine sarcoids: An in vitro study. Res. Vet. Sci..

[115] Mills P.C., Cross S.E. (2007). Regional differences in transdermal penetration of fentanyl through equine skin. Res. Vet. Sci..

[116] Ferrante M., Andreeta A., Landoni M.F. (2010). Effect of different penetration enhancers on diclofenac permeation across horse skin. Vet. J..

[117] Gorzelanny C., Mess C., Schneider S.W., Huck V., Brandner J.M. (2020). Skin Barriers in Dermal Drug Delivery: Which Barriers Have to Be Overcome and How Can We Measure Them?. Pharmaceutics.

[118] Riviere J.E., Papich M.G. (2001). Potential and problems of developing transdermal patches for veterinary applications. Adv. Drug Deliv. Rev..

[119] Mansmann R.A., Currie M.C., Correa M.T., Sherman B., vom Orde K. (2011). Equine behaviour problems—Around Farriery: Foot pain in 11 horses. Equine Vet. Sci..

[120] Melotti L., Carolo A., Elshazly N., Boesso F., Da Dalt L., Gabai G., Perazzi A., Iacopetti I., Patruno M. (2022). Case Report: Repeated Intralesional Injections of Autologous Mesenchymal Stem Cells Combined With Platelet-Rich Plasma for Superficial Digital Flexor Tendon Healing in a Show Jumping Horse. Front. Vet. Sci..

[121] Lorello O., Orsini J.A., Orsini J.A., Divers T.J. (2014). Chapter 3—Intravenous Catheter Placement. Equine Emergencies.

[122] Chapman A. (2017). Placement and Care of Intravenous Catheters. Manual of Clinical Procedures in the Horse.

[123] McCall C. (2012). How to Give Your Horsean Intramuscular Injection. Horse.

[124] Orsini J.A., Orsini J.A., Divers T.J. (2014). Chapter 4—Venous Access via Cutdown. Equine Emergencies.

[125] Sellnow L. (1997). Skin Diseases in Horses. Horse.

[126] Me V. (2009). Variation of Skin Thickness over the Equine Body and the Correlation between Skin Fold Measurement and Actual Skin Thickness. Utrecht University Student Theses Repository. https://studenttheses.uu.nl/handle/20.500.12932/4003.

[127] Mishra D.K., Pandey V., Maheshwari R., Ghode P., Tekade R.K., Tekade R.K. (2019). Chapter 15—Cutaneous and Transdermal Drug Delivery: Techniques and Delivery Systems. Basic Fundamentals of Drug Delivery.

[128] Mills P.C., Magnusson B.M., Cross S.E. (2003). Effect of solute lipophilicity on penetration through canine skin. Aust. Vet. J..

[129] Puschmann T., Ohnesorge B. (2015). Complications After Intramuscular Injections in Equids. Equine Vet. Sci..

[130] Pratanaphon R., Akesowan S., Khow O., Sriprapat S., Ratanabanangkoon K. (1997). Production of highly potent horse antivenom against the Thai cobra (Naja kaouthia). Vaccine.

[131] Van Donkersgoed J., VanderKop M., Salisbury C., Sears L., Holowath J. (1999). The effect of administering long-acting oxytetracycline and tilmicosin either by dart gun or by hand on injection site lesions and drug residues in beef cattle. Can. Vet. J..

[132] Giguère S., Sturgill T.L., Berghaus L.J., Grover G.S., Brown S.A. (2011). Effects of two methods of administration on the pharmacokinetics of ceftiofur crystalline free acid in horses. J. Vet. Pharmacol. Ther..

[133] Steffey E.P., Zinkl J., Howland D. (1979). Minimal changes in blood cell counts and biochemical values associated with prolonged isoflurane anesthesia of horses. Am. J. Vet. Res..

[134] Wagner A.E., Hellyer P.W. (2000). Survey of anesthesia techniques and concerns in private veterinary practice. J. Am. Vet. Med. Assoc..

[135] Gómez de Segura I.A., De Rossi R., Santos M., López San-Roman J., Tendillo F.J., San-Roman F. (1998). Epidural Injection of Ketamine for Perineal Analgesia in the Horse. Vet. Surg..

[136] Howard R., McIlwraith C. (1993). Sodium hyaluronate in the treatment of equine joint disease. Compend. Contin. Educ. Pract. Vet..

[137] García M., Monge M.a., León G., Lizano S., Segura E., Solano G., Rojas G., Gutiérrez J.M.a. (2002). Effect of Preservatives on IgG Aggregation, Complement-activating Effect and Hypotensive Activity of Horse Polyvalent Antivenom Used in Snakebite Envenomation. Biologicals.

[138] Petit A., Redout E.M., van de Lest C.H., de Grauw J.C., Müller B., Meyboom R., van Midwoud P., Vermonden T., Hennink W.E., René van Weeren P. (2015). Sustained intra-articular release of celecoxib from in situ forming gels made of acetyl-capped PCLA-PEG-PCLA triblock copolymers in horses. Biomaterials.

[139] Johnston G.C.A., Wood K.A., Jackson K.V., Perkins N.R., Zedler S.T. (2020). Evaluation of the inflammatory response to two intra-articular hyaluronic acid formulations in normal equine joints. J. Vet. Pharmacol. Ther..

[140] Rhodin M., Haubro Andersen P., Holm Forsström K., Ekstrand C. (2022). In vivo joint synovial fluid disposition of a novel sustained-release formulation of diclofenac and hyaluronic acid in horses. J. Vet. Pharmacol. Ther..

[141] Sykes B.W., Kathawala K., Song Y., Garg S., Page S.W., Underwood C., Mills P.C. (2017). Preliminary investigations into a novel, long-acting, injectable, intramuscular formulation of omeprazole in the horse. Equine Vet. J..

[142] Dowling P., Chirino-Trejo M. (1999). Long-acting oxytetracycline–polyethylene glycol in horses: Pharmacokinetics and tolerance. AAEP Proc..

[143] Baggot J.D. (1988). Veterinary drug formulation for animal health care: An overview. J. Control. Release.

[144] Rathbone M., Brayden D. (2009). Controlled release drug delivery in farmed animals: Commercial challenges and academic opportunities. Curr. Drug Deliv..

[145] Rezende M.L., Boscan P., Stanley S.D., Mama K.R., Steffey E.P. (2010). Evaluation of cardiovascular, respiratory and biochemical effects, and anesthetic induction and recovery behavior in horses anesthetized with a 5% micellar microemulsion propofol formulation. Vet. Anaesth. Analg..

[146] Ahmed I., Kasraian K. (2002). Pharmaceutical challenges in veterinary product development. Adv. Drug Deliv. Rev..

[147] Pirie R.S., Mueller H.-W., Engel O., Albrecht B., von Salis-Soglio M. (2021). Inhaled ciclesonide is efficacious and well tolerated in the treatment of severe equine asthma in a large prospective European clinical trial. Equine Vet. J..

